# Safety of Fibrinogen Concentrate in Non-Trauma and Non-Obstetric Adult Patients during Perioperative Care: Systematic Review and Meta-Analysis

**DOI:** 10.3390/jcm13123482

**Published:** 2024-06-14

**Authors:** Manuela Gomes, Miguel Ângelo-Dias, Gonçalo S. Duarte, Sara Simões Dias, Sofia Silvério Serra, Jorge Lima

**Affiliations:** 1 Transfusion Medicine Department, Hemovida, Hospital da Luz Lisboa, Luz Saúde, 1500-650 Lisboa, Portugal; amdrgv@gmail.com; 2 NOVA Medical School (NMS), Faculdade de Ciências Médicas (FCM), Universidade Nova de Lisboa, 1099-085 Lisboa, Portugal; miguel.dias@nms.unl.pt; 3 Comprehensive Health Research Centre (CHRC), NOVA Medical School (NMS), Faculdade de Ciências Médicas (FCM), Universidade Nova de Lisboa, 1099-085 Lisboa, Portugal; sara.dias@nms.unl.pt; 4 Clinical Pharmacology, Hospital da Luz Lisboa, Luz Saúde, 1500-650 Lisboa, Portugal; gduarte@edu.ulisboa.pt; 5 Laboratory of Clinical Pharmacology and Therapeutics, Faculty of Medicine, University of Lisbon, 1649-004 Lisboa, Portugal; 6 ciTechCare—Centre for Innovative Care and Health Technology, Polytechnic of Leiria, 2411-901 Leiria, Portugal; 7 Library, NOVA Medical School (NMS), Faculdade de Ciências Médicas (FCM), Universidade Nova de Lisboa, 1099-085 Lisboa, Portugal; sofia.serra@nms.unl.pt; 8 Department of Obstetrics and Gynecology, Hospital da Luz Lisboa, Luz Saúde, 1500-650 Lisboa, Portugal

**Keywords:** fibrinogen concentrate, perioperative care, hypofibrinogenaemia, meta-analysis, systematic review

## Abstract

**Background**: Low fibrinogen levels are associated with an increased risk of perioperative bleeding. However, there is an ongoing debate over the ideal treatment threshold, the benefits of prophylactic supplementation with fibrinogen concentrate, and the best source of fibrinogen. While fibrinogen concentrate supplementation is being widely used to treat bleeding related to acquired haemostatic deficiencies, there is a lack of evidence regarding its dosage, effectiveness, and safety. This systematic review provides an up-to-date summary of the relationship between fibrinogen concentrate supplementation and safety measures in the perioperative care of non-trauma, non-obstetric adult patients. **Methods**: A comprehensive online search was conducted on PubMed/Medline, EMBASE, Scopus, Web of Science, the Cochrane Database of Systematic Reviews, and the Cochrane Central Register of Controlled Trials. **Results**: This systematic review and meta-analysis encompasses ten studies involving 1391 patients. There was a decreased risk of total thromboembolic events in patients treated with fibrinogen compared to the control (OR 0.65, 95% CI 0.43 to 0.98, I^2^ = 0%). In addition, when fibrinogen was used prophylactically, it resulted in shorter ICU stays (MD −1.50, 95% CI −2.64 to −0.36), when set against its therapeutic use. A sensitivity analysis on cardiovascular surgery studies did not reveal any statistically significant difference. **Conclusions**: The use of fibrinogen concentrate in the perioperative care of non-trauma and non-obstetric adult patients may lead to potential benefits.

## 1. Introduction

Surgery may lead to severe bleeding, which can cause haemorrhagic shock—a potentially fatal condition marked by tissue hypoxia, cellular ischaemia, and eventual organ dysfunction, potentially leading to organ failure and death [1]. Fibrinogen, also known as clotting factor I, is a liver-produced glycoprotein vital to both primary and secondary haemostasis. When thrombin cleaves it, fibrinogen produces soluble fibrin monomers, which polymerise to create a clot base and promote platelet aggregation through binding to glycoprotein IIb/IIIa receptors on platelets. Consequently, fibrinogen proves critical for effective blood clotting and is the first factor drained in cases of extreme bleeding and haemodilution, a situation worsened by hypothermia and acidosis [2]. Researchers link declining fibrinogen levels during bleeding to increased morbidity and mortality rates across various clinical situations, including trauma-induced coagulopathies [3,4,5]. Unsurprisingly, given fibrinogen’s distinct role in sustaining haemostasis, its administration has proven consistently effective in animal models and patients with acquired fibrinogen deficiency, becoming a critical step to control blood loss in critical settings [6]. In these situations, standard coagulation tests, or, preferably, viscoelastic testing, can corroborate clinical suspicions of a fibrinogen deficit. Normal plasma levels extend from 1.5 to 4.5 g·L^−1^ but can naturally be higher [7,8].

Fibrinogen concentrate (FC) is a widely used haemostatic agent in many countries, with several decades of pharmacovigilance yielding relatively few reports of adverse events [9], but fibrinogen replacement is still a matter of debate for several reasons. Firstly, when it comes to supplementation, there are three options: plasma, cryoprecipitate, and FC. Plasma is not the best source for fibrinogen replacement, whereas cryoprecipitate has variable fibrinogen levels and contains other coagulation factors. FC, however, is advantageous since it has a fixed amount of fibrinogen in a small volume (1 g per vial, potentially ranging from 0.9 g to 1.3 g), undergoes pasteurisation and viral inactivation, and is readily available [10,11]. However, FC may not be available in all countries for treating acquired coagulopathies. The suggested doses to correct hypofibrinogenaemia in bleeding patients with coagulation disorders range from 25 to 75 mg·kg^−1^, dependent upon various factors, including the outcome of viscoelastic testing or the Clauss method [12]. Secondly, selection and target levels bring another layer of discussion and appear to rely on different factors and clinical circumstances [13]. Lastly, FC is a procoagulant given to patients with coagulation disorders who already have accumulated risk factors for developing thrombotic or thromboembolic episodes. Although numerous clinical trials suggest that FC is effective and seemingly safe, reliability is curtailed by the vast heterogeneity and low quality of the studies conducted, and their small sample sizes, limiting the rigorous recommendation of FC use in minimising blood transfusion requirements [14,15,16].

Several published review studies have offered an insight into the impact of FC on bleeding patients, but there are noteworthy limitations. Firstly, meta-analyses by Fominskiy and Wikkelso [14,16] include both paediatric and adult populations, which could skew findings, as fibrinogen levels are known to vary with age [17]. Secondly, Fominskiy et al.’s [16] inclusion of a post-partum haemorrhage study could add heterogeneity, due to pregnancy-related alterations in fibrinogen levels. Both these meta-analyses also include trauma patients whose bleeding is influenced by multiple factors such as tissue injury and shock, thereby potentially skewing mortality outcomes and affecting the reliability of conclusions [5,18]. In the third place, despite Ka Ting et al.’s meta-analysis [15] intending to minimise such heterogeneity, its search databases may not have been comprehensive enough, possibly resulting in overlooked pertinent studies. Lastly, other systematic reviews with meta-analyses include limited studies or only focus on specific clinical settings, surgeries, and comparators, thus lacking a comprehensive evaluation of FC effectiveness and safety in bleeding patients [19,20,21].

Future studies need to meticulously gather and analyse all existing data on FC in patients with acquired hypofibrinogenaemia who bleed. It is necessary to thoughtfully choose suitable populations and comparators and consider the flaws of prior studies to obtain more precise and dependable conclusions. This systematic review primarily aimed to explore the association between safety outcomes in adult patients—excluding obstetric and trauma cases—who underwent cardiovascular, abdominal, or orthopaedic surgery and received fibrinogen replacement during the perioperative phase.

## 2. Materials and Methods

This study was preregistered in PROSPERO (No: CRD42023395383) and conducted following the guidelines of the Preferred Reporting Items for Systematic Review and Meta-Analysis (PRISMA) statement [22,23] (online Supporting Information Table S1).

### 2.1. Information Sources and Search Strategy

On 3 March 2023, a comprehensive computerised literature search was conducted across various electronic databases, namely PubMed/Medline, EMBASE, Scopus, Web of Science, the Cochrane Database of Systematic Reviews, and CENTRAL. We used a search strategy involving database-specific subject heading terms along with their free-text word variants, accounting for the unique features of each database (online Supporting Information Table S2). These were then combined using the Boolean operators ‘OR’ and ‘AND’. The search was limited to studies involving human adults; there were no restrictions on the publication date or language. In addition, a manual review of the references from the chosen studies was carried out to find any pertinent publications that might have been missed. The search was updated in December 2023, and no additional eligible articles were found.

Rayyan Intelligent Systematic Review software was utilised to store, organise, and manage all references resulting from the literature search [24].

### 2.2. Eligibility Criteria

The inclusion criteria were as follows: the study must be an RCT; the population must consist of patients undergoing surgical care in cardiovascular, abdominal, or orthopaedic surgeries who are experiencing bleeding during the perioperative period; the intervention must involve FC supplementation in comparison to standard treatments, placebos, or other haemostatic agents; and the outcomes must evaluate the safety of FC supplementation.

The exclusion criteria were as follows: studies involving patients with hereditary bleeding disorders leading to fibrinogen deficiency or abnormal function were excluded, as were studies focusing on trauma or obstetric patients, as these were deemed incorrect populations. Studies presented only in abstract form without accompanying full-text publications, individual case studies, editorials, commentaries, letters to the editor, and review articles were not included, as these were considered the wrong publication type. Duplicate studies were also excluded.

### 2.3. Study Selection

Two authors (MG, MAD) independently screened the titles and abstracts, with any conflicts resolved by a third reviewer (JL). The remaining studies were full text examined by two authors (MG, MAD), and selection was based on the eligibility criteria. In case of disagreements, a third reviewer (JL) was consulted to achieve consensus. All decisions, as well as reasons for exclusion, were recorded. The number of articles chosen at each stage is depicted in a flow chart in compliance with the PRISMA 2020 guidelines. The review authors had access to information about journal titles, study authors, and their institutions.

### 2.4. Data Collection

Two independent reviewers (MG, MAD) extracted all pertinent data from each chosen study. Only relevant information from studies assessing multiple outcomes and variables was collected for this review. For missing or unreported data, the respective authors of the included studies were contacted for clarification. Any disagreements were resolved by consulting a third reviewer (JL). Using an extraction form, two authors (MG, MAD) independently extracted all relevant data from each study, namely, the characteristics of the study (title, authors, year of publication, journal, country of origin, study design, number of participants and their characteristics, and recruitment procedure and duration), characteristics of intervention (indication, timing, type of surgery, criteria for FC administration, dose of FC administered, and type and dose of comparator), and outcomes.

Data were recorded using Microsoft Excel^TM^ [25].

### 2.5. Outcomes

The primary outcome was overall mortality. To analyse this outcome, we used the data from the longest follow-up period reported for mortality in each study, regardless of the specific duration across the studies. This period did not exceed 30-day mortality in any of the studies. Secondary outcomes were composite adverse and serious adverse events, and specific adverse events associated with fibrinogen concentrate intervention, namely, thromboembolic events, myocardial infarction, deep venous thrombosis or pulmonary embolism, stroke or transient ischaemic attack, the total length of stay in hospital, and the length of stay in the ICU. Adverse events and serious adverse events were defined by each study’s authors and observed and assessed by safety outcome assessors in the operating theatre or postanaesthetic care unit, or during the follow-up period after surgery, depending on each study. The risk of each outcome was measured as the number of patients experiencing a given outcome. This review followed a confirmatory approach, as pre-specified in the protocol, in which anticipated or already recognised specific adverse events associated with the intervention and assumed to be measured regularly and consistently in studies were chosen [26].

Only studies reporting outcome data of interest were considered eligible. We did not extract data as ‘zero’ unless they were clearly listed as such in the report. No interpretation of withdrawals as surrogate markers for safety or tolerability was adopted [26].

### 2.6. Risk of Bias

The Cochrane risk of bias tool was utilised to evaluate the methodological quality of each study independently [27,28]. A second reviewer (GSD) examined this evaluation, with any disagreements settled by a third independent reviewer (MG). This tool categorises studies into a low, high, or unclear risk of bias in areas such as random sequence generation, allocation concealment, blinding of participants and personnel, blinding of outcome assessment, incomplete outcome data, and selective reporting [27,28]. We also checked for industry sponsorship or conflicts of interest.

### 2.7. Statistical Analysis

We utilised R statistical software (version 4.3.0) and package metafor [29] for our statistical analysis and the creation of forest plots. The pooling of our data was executed using a random effects model, given the anticipated heterogeneity in the included trials, mainly the differences in study design. We used an empirical Bayes estimator to pool data. We reported pooled dichotomous data through odds ratios (ORs) and mean difference (MD) for continuous data, with 95% confidence intervals (95% CIs) for both. When trials presented continuous data as the median with IQR, the values were converted to the mean and SD, following the Cochrane methodology [30]. Heterogeneity was examined using tau^2^ and I^2^ [31]. Subgroup analyses were conducted, considering the comparator group (cryoprecipitate, platelets, placebo, or no treatment) and whether the intervention was prophylactic or therapeutic. Post hoc sensitivity analyses were performed for all outcomes by including only data from cardiovascular surgery trials.

For the analysis of publication bias, we conducted a linear regression of funnel plot asymmetry using Egger’s test [32]. Statistical significance was considered at *p* < 0.05.

## 3. Results

### 3.1. Study Selection

The database search yielded 2542 studies, from which 653 were dismissed as duplicates. We found an additional seven studies by a manual search of references lists. After screening the remaining 1882 records by title and abstract, we excluded 1818 studies that evidently did not meet our eligibility criteria. We identified 64 potentially relevant records for a full review. Eventually, we acquired and examined 59 full-text articles, only to exclude another 49 due to inapplicable publication type (26 records), non-RCT studies (15 records), irrelevant outcomes (5 records), or a not-of-interest study population (3 records).

**Figure 1:**
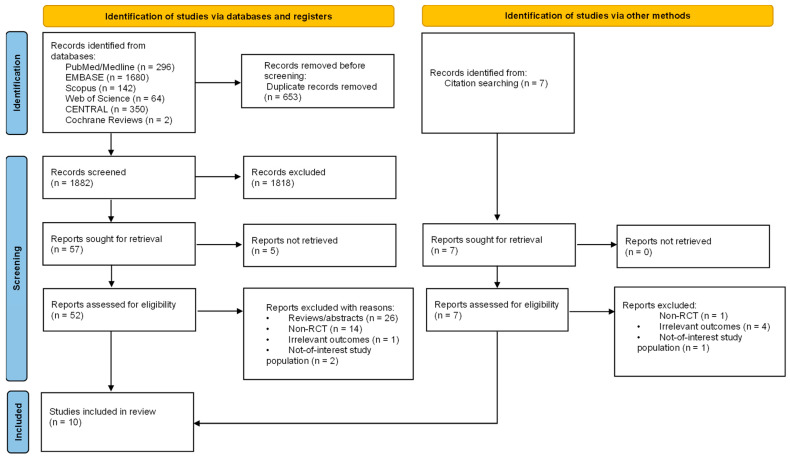
PRISMA 2020 flow diagram of systematic review process and study selection.

Ten studies fulfilled all criteria and were selected for systematic review and meta-analysis [33,34,35,36,37,38,39,40,41,42]. Figure 1 illustrates the process of study selection.

### 3.2. Characteristics of Included Studies

Table 1 summarises the features of the included studies. These articles, published from 2009 to 2019, originate from 16 different countries.

Among the studies, seven focused on cardiovascular surgery [33,34,35,39,40,41,42], one on elective liver transplantation [37], one on cytoreductive surgery [36], and one on hip arthroplasties [38]. The goal of evaluating FC administration in treating bleeding patients varied across the trials. Four studies examined the advantages of administering FC as a first-line treatment pre-operation or during surgery, to prevent bleeding and reduce postoperative blood loss and transfusion needs [37,38,39,40]. Three studies evaluated the benefits of using a single intra-operative therapeutic bolus of FC to control bleeding after complicated cardiac surgery, without selection based on plasma FC level [34,35,42]. Three studies aimed to juxtapose therapeutic FC administration with other haemostatic agents to treat acquired hypofibrinogenaemia [33,36] or poor haemostatic conditions like moderate to severe bleeding [41].

The criteria for FC administration varied across the trials reviewed. Three studies used specific plasma fibrinogen levels as criteria: Karlsson [39] set the limit at pre-operative fibrinogen ≤3.8 g·L^−1^, Sabate [37] used pre-operative fibrinogen ≤ 2.9 g·L^−1^ or intra-operative levels < 1 g·L^−1^, while Callum [33] considered intra-operative fibrinogen < 2.0 g·L^−1^ or the clot amplitude at 10 min < 10 mm by thromboelastometry as criteria. Three other studies applied a bleeding mass criterion, specifically 60–250 g [34,35] or 60–250 mL [42] within the first 5 min after removal from cardiopulmonary bypass (CPB). One trial administered FC according to predicted blood loss during surgery; FC was administered when the blood loss was projected to surpass 2 L when 90 min into the procedure [36]. Another study started FC administration when the visible bleeding scale reached 2 (controllable bleeding with applied pressure) or 3 (multiple diffuse bleeding sites) [41]. Two remaining studies did not establish any predefined criteria for FC administration [38,40].

Three studies utilised set doses of FC at 2 g and 4 g [33,39,41], one study used a fibrinogen dose determined by body weight (30 mg fibrinogen·kg^−1^) [38], and another determined by plasma fibrinogen levels at the end of CPB and body weight, and corrected for haematocrit [42]. Three additional studies figured the fibrinogen dose based on the ROTEM/FIBTEM maximum clot firmness parameter [34,35,40]. One study administered 1 g of fibrinogen, aiming to reach a plasma value of 2.9 g·L^−1^ [37], and a different study began with a 4 g dose of FC. If the FIBTEM A20 parameter remained ≤12 mm, they supplemented it with a further 4 g dose [36].

Two studies utilised Octapharma’s Fibryga^®^ [33,36], while eight others employed CSL Behring’s Haemocomplettan^®^/RiaSTAP^®^ [34,35,37,38,39,40,41,42] as FC products. Six studies compared the results with a placebo, either isotonic saline [34,35,37,38,40] or saline solution with albumin [42]; two used cryoprecipitate for comparison [33,36]; one compared with apheresis platelets [41]; and one did not provide any treatment for comparison [39].

The study included 702 patients in the intervention group and 689 in the comparison group. Sample sizes ranged from 10 to 372 patients.

### 3.3. Risk of Bias

Table 2 displays the risk of bias assessment results of the included studies.

Seven studies used a computer-generated method for random sequence allocation [33,34,35,37,38,40,42], while three did not disclose the method used [36,39,41]. Almost every selected study adequately concealed allocation (usually with unmarked, sealed opaque envelopes or syringes), with one not giving this information [38].

The blinding practices used varied across the studies we reviewed. Five studies ensured that participants, caregivers, and outcome assessors were adequately blinded [34,35,37,40,42]. In one study, the clinicians giving the treatment were not blinded; however, participants, data collectors, and outcome assessors were blinded [33]. Two studies did not specifically mention if the participants were blinded, but all other involved staff and outcome assessors were [37,39]. Another study acknowledged that clinician blinding was impractical during surgeries due to noticeable differences in study drugs [36]. One study was conducted as open-label [41] and another as single-blind [38].

Four studies addressed intention-to-treat analyses and covered methods for handling missing data [34,35,37,42]. One study reported that participants were lost during follow-up or removed after randomisation, but the missing data were proportionate across groups, and the reasons were unrelated to the outcome [33]. Another study removed two participants (4.4%), each from a different group, due to significant protocol deviations (the FC group was underdosed, and the comparison group was overdosed) [36]. In four studies, no participants were excluded following randomisation [38,39,40,41].

Concerning selective reporting bias, one study failed to report an outcome specified in their protocol—namely, the volume of transfused packed red blood cells during and post-surgery [38]. Another study did not provide a trial registration or protocol number in its manuscript [39]. There were no suspicions of selective reporting in the other studies, as each outcome reported adhered to the established protocols.

Nine trials were sponsored by the respective FC supplier. One study did not provide such information [38]. Five studies disclosed conflicts of interest [33,34,35,36,40], three studies stated no conflict of interest [37,41,42], and two studies did not provide such information [38,39].

Overall, three out of the ten trials included were deemed to have a high risk of bias. This was due to the lack of assurance that the participants and personnel in these three trials were blinded to the received intervention [36,38,41].

### 3.4. Thromboembolic Events

**Figure 2:**
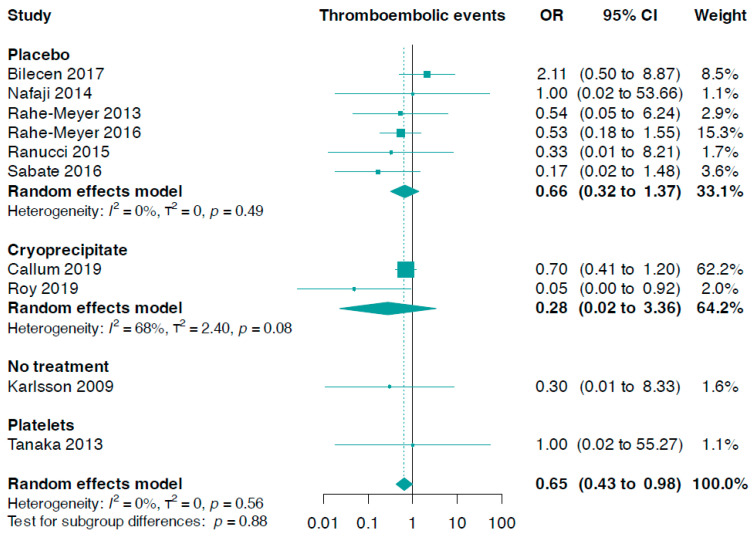
Forest plots for thromboembolic events according to comparator group [33,34,35,36,37,38,39,40,41,42].

Data from ten trials (n = 1391) were included in this analysis. The combined analysis revealed that patients treated with fibrinogen had a reduced risk of total thromboembolic events compared to the control (OR 0.65, 95% CI 0.43 to 0.98, I^2^ = 0%, Figure 2). A subgroup analysis considering the control group did not yield statistically significant results (interaction *p*-value = 0.88).

Additionally, we found no statistically significant difference between fibrinogen and controls in stroke or transient ischaemic attack (OR 0.91, 95% CI 0.51 to 1.60, I^2^ = 0%, six trials, n = 1214, online Supporting Information Figure S1). This also applied to myocardial infarction (OR 0.98, 95% CI 0.37 to 2.61, I^2^ = 0%, seven trials, n = 1102, online Supporting Information Figure S2), as well as deep venous thrombosis or pulmonary embolism (OR 0.55, 95% CI 0.24 to 1.26, I^2^ = 0%, nine trials, n = 1371, online Supporting Information Figure S3).

**Figure 3:**
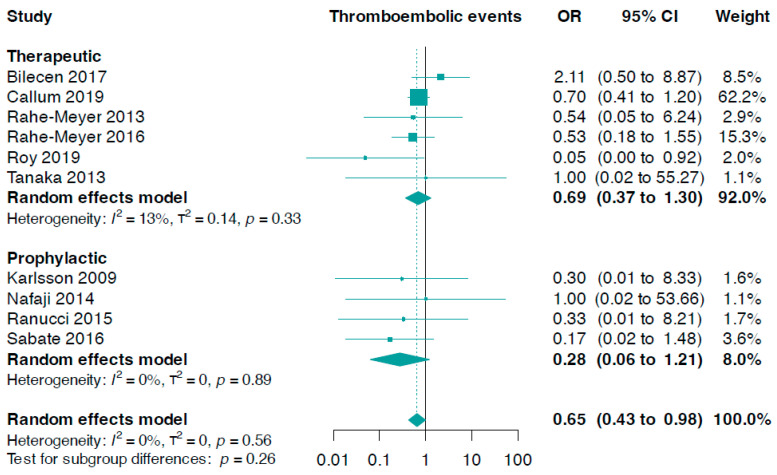
Forest plots for thromboembolic events according to indication setting [33,34,35,36,37,38,39,40,41,42].

The subgroup analysis, which focused on fibrinogen administration indications, did not produce statistically significant results (*p*-value for interaction = 0.26, Figure 3). We discovered that prophylactic fibrinogen use (OR 0.28, 95% CI 0.06 to 1.21, I^2^ = 0%) was not different from its therapeutic use (OR 0.69, 95% CI 0.37 to 1.30, I^2^ = 13%) in diminishing the risk of thromboembolic events (Figure 3). In addition, there were no notable differences concerning the risk of stroke or transient ischaemic attack (*p*-value for interaction = 0.94, online Supporting Information Figure S4), myocardial infarction (*p*-value for interaction = 0.62, online Supporting Information Figure S5), and deep venous thrombosis or pulmonary embolism events (*p*-value for interaction = 0.60, online Supporting Information Figure S6).

We also conducted a linear regression of funnel plot asymmetry using Egger’s test, which did not indicate evidence of publication bias for thromboembolic events (*p*-value = 0.125).

### 3.5. Mortality

**Figure 4:**
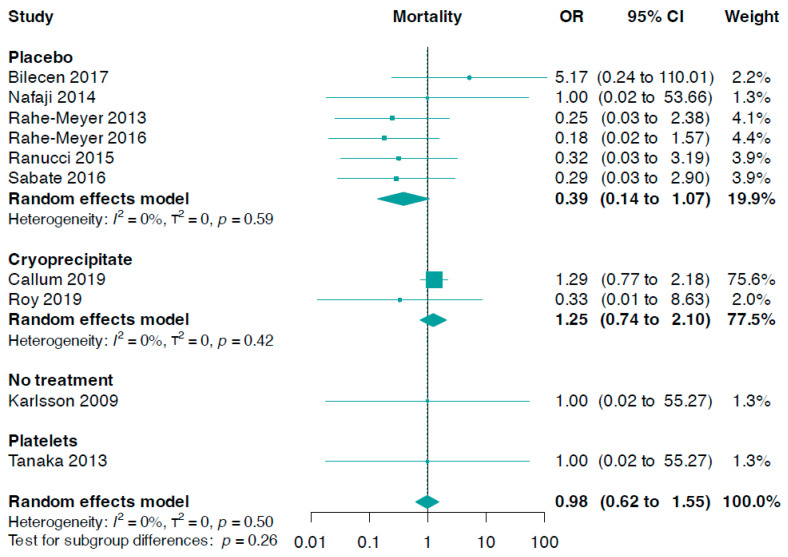
Forest plots for mortality according to comparator group [33,34,35,36,37,38,39,40,41,42].

Data for this outcome were contributed by ten trials (n = 1391). The pooled analysis indicated there was no meaningful statistical difference between fibrinogen and controls (OR 0.98, 95% CI 0.62 to 1.55, I^2^ = 0%, Figure 4). However, no significant statistical difference was observed from a subgroup analysis based on the control group (*p*-value for interaction = 0.26). Nevertheless, fibrinogen usage showed a slightly, but not statistically significant, lower mortality in comparison to a placebo (OR 0.39, 95% CI 0.14 to 1.07, I^2^ = 0%). In addition, there were no significant statistical differences in comparisons of fibrinogen versus cryoprecipitate, fibrinogen versus platelets, or fibrinogen versus no treatment (Figure 4).

The subgroup analysis, based on the need for fibrinogen administration, was not statistically significant (interaction *p*-value = 0.36, online Supporting Information Figure S7).

We also conducted a linear regression of funnel plot asymmetry using Egger’s test, which did not indicate evidence of publication bias for overall mortality (*p*-value = 0.074).

### 3.6. Length of Hospital Stay

The combined analysis did not reveal a meaningful statistical discrepancy in hospital stays between fibrinogen and controls (MD −0.61, 95% CI −1.36 to 0.14, I^2^ = 0%, five trials, n = 839, online Supporting Information Figure S8), nor in ICU duration (MD −0.48, 95% CI −1.13 to 0.18, I^2^ = 48%, five trials, n = 912, online Supporting Information Figure S9).

**Figure 5:**
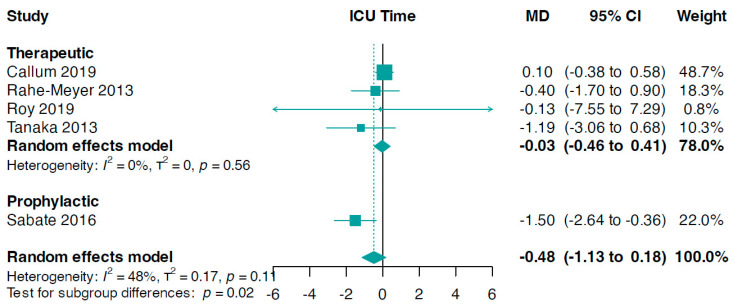
Forest plots for ICU time according to indication setting. MD, mean difference [33,34,36,37,41].

A subgroup analysis based on the reasons for fibrinogen administration revealed significant results (interaction *p*-value = 0.02, Figure 5). The data showed that the prophylactic use of fibrinogen resulted in a reduction in ICU stay duration (MD −1.50, 95% CI −2.64 to −0.36) compared to the therapeutic use of fibrinogen (MD −0.03, 95% CI −0.46 to 0.41, I^2^ = 0%, Figure 5).

### 3.7. Safety Outcomes

**Figure 6:**
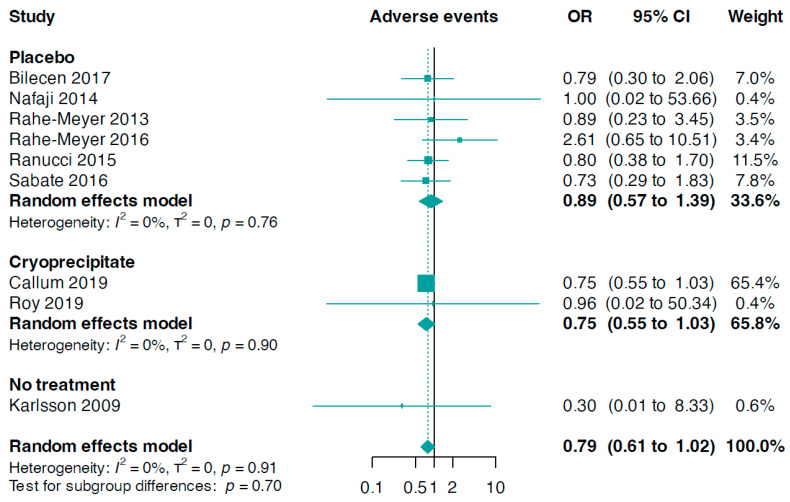
Forest plots for adverse events according to comparator group [33,34,35,36,37,38,39,40,42].

Data for this outcome were contributed by nine trials (n = 1371). The combined analysis revealed a slightly, but not statistically significant, reduced risk of composite adverse events in patients treated with fibrinogen compared to the control group (OR 0.79, 95% CI 0.61 to 1.02, I^2^ = 0%, Figure 6). In addition, there was no significant statistical difference between the fibrinogen group and the control group concerning serious adverse events (OR 0.65, 95% CI 0.37 to 1.16, I^2^ = 53%, five trials, n = 1085, online Supporting Information Figure S10).

The subgroup analysis, depending on the control group or the reason for administering fibrinogen (online Supporting Information Figures S11 and S12), did not yield statistically significant results.

### 3.8. Sensitivity Analysis on Cardiovascular Studies

Since most of the included trials were on cardiovascular surgery (seven trials, n = 1224), we performed a sensitivity analysis for all outcomes focusing exclusively on these trials. However, we found no statistically significant differences for any of the outcomes investigated (online Supporting Information Table S3)

## 4. Discussion

Excessive blood loss during and after surgery is a common, critical complication. It can lead to extended stays in the ICU, increased reliance on mechanical ventilation, heightened health risks and death rates, and elevated healthcare costs [43,44]. Fibrinogen, a vital component for achieving and maintaining haemostasis, significantly decreases during bleeding [6]. As a result, FC is increasingly used worldwide to control bleeding in patients with acquired hypofibrinogenaemia. However, robust evidence supporting its safety is still limited.

Our systematic review of ten studies involving 1391 patients highlights a decreased overall risk of thromboembolic events after administering FC in perioperative settings, and a reduction in ICU stay when used prophylactically. Additionally, although our analysis showed a small and potentially beneficial effect of FC intervention on overall mortality, particularly when compared to a placebo, and a reduced risk of composite adverse events, these observations were not statistically significant. Thus, more trials are needed to substantiate these findings. Nevertheless, these observations are crucial for haemostatic agents in light of past cases where products like Factor VIIa effectively minimised blood loss but raised safety issues due to thrombosis and mortality risks [45,46]. To our understanding, this study offers the most thorough and current meta-analysis, focusing on the safety of FC use in specific non-trauma, non-obstetric adult patients during perioperative care.

In this study, we chose to analyse the risk of adverse events, to achieve a balanced perspective, as these often take second place in the evaluation of interventions and may make the intervention look more favourable than it should. However, the monitoring and reporting of adverse events in a clinical trial is a complex task, involving various considerations. This includes ensuring the proper blinding of both patients and investigators, distinguishing between adverse and serious adverse events, attributing adverse events to study drugs, collecting accurate reports from patients, and maintaining consistent, transparent monitoring, coding, and reporting by investigators. In addition, some adverse effects occur rarely or may become apparent long after the start of the intervention. This contrasts with adverse events that have a higher incidence and occur soon after the intervention is delivered. Therefore, it is important to recognise that factors such as the duration of follow-up and the selection criteria in randomised trials may exclude participants at an increased risk of harm.

Adverse events are seldom specified as primary outcomes or are not pre-specified at all, so there is often a lack of clarity in the methods used to obtain adverse events data. The trials included in this review employed varied approaches to define, monitor, and report adverse events. Some trials coded composite adverse and serious adverse events in a systematic way using the Medical Dictionary for Regulatory Activities (MeDRA) [33,36]. In one study, composite and serious adverse events were provided, along with the criteria used for their definition [37], while another provided only data on specific adverse events, leaving uncertainty about the occurrence of other adverse events [42]. Some trials reported several categories of adverse events without providing clear definition of each [34,35], others reported only total adverse events [38,39,40], and one trial did not provide any data on adverse events [41].

Adverse events may not necessarily be linked to the intervention product, even when they are known to be associated with that category of drug. Some of the included trials specifically addressed causality between adverse events and study drugs by presenting data on both adverse events observed regardless of relation to the study drug, and adverse events possibly, probably, or definitely related to the study drug [34,35,40], while other trials addressed this issue but did not provide such data [37], or they did not address this issue at all [33,38,39,41,42].

Most of the included trials presented all observed adverse events, while bigger trials presented the most frequently occurring adverse events [33,35]. Finally, none of the included studies were designed to address safety as the primary outcome. Furthermore, we also decided to include reported symptoms related to drug administration when they were not specifically labelled as adverse events, in order not to potentially dismiss good-quality data due to a lack of correct phrasing. Finally, we performed meta-analyses of both composite and specific adverse events [47], though we are aware of the difficulties in interpreting composite adverse outcomes that are potentially constructed from several diverse events, since an important signal of rare serious adverse events could be masked by common trial adverse events [26].

Our study is based solely on published data due to practical constraints, acknowledging that information taken from published reports may be incomplete or lack specificity [48]. This limitation arises from challenges in accessing unpublished information, introducing the possibility of discordant findings. Of note, our analysis did not reveal signs of publication bias, suggesting that the observed reduction in thromboembolic events for the fibrinogen arm was not disproportionately influenced by selectively reported positive outcomes. While the exclusion of non-published data is a limitation, the absence of publication bias adds credibility to our findings, emphasizing the need for future research to address this limitation and enhance the overall robustness of conclusions in this field. To the best of our knowledge, a search of trial registries yielded no ongoing studies assessing safety outcomes in non-obstetric and non-trauma adult patients who underwent cardiovascular, abdominal, or orthopaedic surgery and received fibrinogen replacement during the perioperative phase.

Our data showed that the prophylactic use of fibrinogen resulted in a reduction in ICU stay duration compared to the therapeutic use of fibrinogen. However, it is crucial to approach these findings with caution, due to the inherent complexities in assessing ICU stay as a safety indicator. Patient characteristics such as age and pre-existing health conditions, variations in clinical practices, and the nature of surgical procedures can all influence the interpretation of these results. Additionally, the impact of evolving medical practices over time underscores the need for a nuanced understanding when evaluating the safety implications of fibrinogen use in perioperative care.

Readers of this review should therefore be mindful of these limitations when critically appraising our data. It is important to remember that association does not imply causality, particularly considering the multitude of factors that contribute to adverse events and outcomes.

This study has numerous strengths. Our review strictly followed a predefined methodology from the Cochrane Handbook for Systematic Reviews of Interventions and was conducted using a previously registered protocol. Additionally, we employed an extensive search strategy using leading bibliographic databases for biomedical research. Unlike other reviews on the topic, we focused exclusively on a specific group of patients, thereby reducing potential bias and the heterogeneity that can result from varied population characteristics.

We acknowledge certain limitations to our study. Some of the included studies had small sample sizes, which may limit the ability to detect rare adverse events. We found a significant number of studies that report on safety outcomes after FC administration, but considerable heterogeneity remains. This includes variances in the countries where the studies took place, data being mainly drawn from cardiac surgery studies, different administration methods adhering to various fibrinogen level thresholds or ROTEM FIBTEM care test targets, and the diverse dosages of FC in all the trials. Such variances could potentially influence the broad applicability and interpretation of our results. Furthermore, the relevance of findings from older studies included in our research could be questionable, due to evolving standards of care and blood management protocols.

In this study, the identified trials comprised patients predominantly undergoing cardiac surgery, but also abdominal and orthopaedic surgeries. We recognise that the inclusion of different types of surgery increases heterogeneity (different clinical contexts, triggers for fibrinogen supplementation, doses of fibrinogen concentrate, and target values), and, due to the limited number of available studies, the applicability of these study findings to other surgical interventions is uncertain. Nevertheless, at a certain point, all surgical patients developed hypofibrinogenaemia, necessitating fibrinogen replacement to control coagulopathy, which may allow for the generalisation of these findings. Thus, the perioperative period can be viewed as a major group of patients, while trauma and postpartum haemorrhage have pathophysiological mechanisms that may imply different triggers and target values for fibrinogen supplementation and/or other haemostatic agents.

We tried to overcome the heterogeneity of the included studies by conducting a sensitivity analysis focusing exclusively on cardiovascular surgery studies, given their predominance within the included trials. However, despite the specificity of the patient population undergoing cardiovascular surgery, fibrinogen administration did not yield significant differences in the risk of total thromboembolic events, overall mortality, length of hospital stays, or any other safety outcomes compared to the control groups. These findings suggest that fibrinogen’s effects may vary substantially across different surgical populations and underscore the need for continued research to elucidate its precise role in clinical practice. Furthermore, we conducted subgroup analyses in order to assess the impact of different comparators and different regimens. However, as the number of included studies is low, the results of these analyses should be interpreted with caution, as the analyses will lack statistical power.

No published systematic reviews with similar results to our current study have been identified. Fominskiy et al. [16] noted a decrease in overall mortality in the FC group, with only four trials contributing to the analysis [34,35,37,40], but detected no difference in the incidence of thrombotic events. A recent review by Ka Ting and associates [15] did not assess mortality and found no significant variations in thromboembolic events using data from only three trials.

In evaluating the safety of any intervention, the assessment of mortality should be considered a mandatory aspect. Despite the possible controversy surrounding mortality as a primary outcome, it is still a crucial safety measure, as it encapsulates both the ultimate harms and benefits of a treatment. Even if participants in the trials included may represent patients with generally low mortality rates, the consideration of this outcome holds significant clinical importance, especially when extrapolating the data to scenarios involving severe, acute bleeding—a common situation in everyday medical practice. Nevertheless, we acknowledge the need for caution when interpreting mortality results from clinical trials. Additionally, not all studies included mortality as a primary endpoint.

Importantly, it is essential to recognise the difficulty in associating a death event solely with an intervention, without considering various contributing factors such as clinical history, surgery risk, age, and complications during and after the operative period. Unfortunately, many studies fail to report whether such events were likely or definitively related to the outcome.

Indeed, the absence of consensus among various published reviews can be attributed to differences in methodologies, clinical settings, age ranges, operational timings, and the reported results [14,15,16,19,20,21]. Additionally, the consideration of two new trials involving 780 patients, which were not included in prior reviews, highlights the ever-changing nature of evidence in this field [33,36].

Although this review features recent studies with larger sample sizes and refined designs, there is still a pressing need for additional trials with well-defined groups of patients. It is crucial to focus on minimising bias and amplifying statistical potency to highlight differences in clinically significant patient safety measures. Extensive multicentre trials during the perioperative period are required to validate the effects of FC on these parameters.

## Figures and Tables

**Table 1:** Characteristics of included studies.

Study	Country	Trial Duration	Intervention Group	Indication	Timing	Type of Surgery	Criteria for FC Administration	Administered FC Dose	Comparator Group	Comparator
Bilecen et al., 2017 [42]	The Netherlands	Feb 2011–Jan 2015	n = 60 70.0 (10.0) yrs 65% males	Therapeutic	Intra- or post-op	Elective high-risk cardiac surgery *	5-min bleeding mass of 60–250 mL immediately after CPB and completion of surgical haemostasis	Fg doses based on plasma Fg levels (Clauss) at end of CPB †. (Haemocomplettan-P^®^, CSL Behring)	n = 60 72.0 (10.0) yrs 73% males	Placebo (2 g of albumin diluted with 0.9% saline solution)
Callum et al., 2019 [33]	Canada	Feb 2017–Nov 2018	n = 372 65 (54–72) yrs 69.6% males	Therapeutic	Intra or post-op	Cardiac with CPB	Plasma Fg < 2.0 g·L^−1^ or FIBTEM– A10 < 10 mm	4 g FC (Fibryga^®^, Octapharma AG)	n = 363 64 (53–72) yrs 71.1% males	10 units of cryoprecipitate (~4 g Fg)
Karlsson et al., 2009 [39]	Sweden	Sep–Dec 2006	n = 10 66 (9) yrs 90% males	Prophylactic	Pre-op	Elective coronary artery bypass graft	Plasma Fg ≤ 3.8 g·L^−1^	2 g FC (Haemocompletan^®^, CSL Behring)	n = 10 68 (8) 90% males	No treatment
Najafi et al., 2014 [38]	Iran	Aug 2011–Feb 2013	n = 15 52.8 (14.9) yrs 46% males	Prophylactic	Intra-op	Total hip arthroplasty	Prophylactic administration	30 mg/kg FC (Haemocomplettan^®^, CSL Behring)	n = 15 51.3 (14.1) yrs 20% males	Placebo (0.9% saline solution)
Rahe-Meyer et al., 2016 [35]	Austria, Brazil, Canada, Czech Republic, Denmark, Finland, Germany, Italy, Japan, Poland, UK	Jan 2012–Sep 2014	n = 78 63.9 (22–86) yrs 76.9% males	Therapeutic	Intra-op	Elective aortic surgery requiring CPB with or without other cardiac surgery	5-min bleeding mass of 60–250 g immediately after CPB and completion of surgical haemostasis	Fg doses determined from MCF of FIBTEM ‡ (Haemocomplettan-P^®^ and RiaSTAP^TM^; CSL Behring)	n = 74 64.2 (24–86) yrs 68.9% males	Placebo (0.9% saline solution)
Rahe-Meyer et al., 2013 [34]	Germany	Jun 2008–Apr 2010	n = 29 59 (14) yrs 66% males	Therapeutic	Intra-op	Elective aortic replacement surgery involving CPB	5-min bleeding mass of 60–250 g immediately after CPB and completion of surgical haemostasis	Fg doses determined from MCF of FIBTEM ‡ (Haemocomplettan-P^®^ and RiaSTAP^TM^; CSL Behring)	n = 32 61 (12) yrs 78% males	Placebo (0.9% saline solution)
Ranucci et al., 2015 [40]	Italy	Nov 2011–2014	n = 58 72 (64–76) yrs 71% males	Prophylactic	Intra-op	Complex cardiac surgery with expected CPB duration ≥ 90 min	Prophylactic administration	FC doses determined on MCF at FIBTEM ‡ (Haemocomplettan^®^, CSL Behring)	n = 58 73 (68–79) yrs 74% males	Placebo (0.9% saline solution)
Roy et al., 2019 [36]	UK	Mar 2017–Jul 2018	n = 22 61 (37–76) yrs 40.9% males	Therapeutic	Intra- or post-op	Cytoreductive surgery for Pseudomyxoma peritonei	Predicted intraoperative blood loss ≥ 2 L 90 min into the surgery	4 g FC (Fibryga^®^, Octapharma AG). Intra- or post-op FIBTEM A20 of ≤12 mm triggered administration of another 4 g/2 g of FC	n = 23 59 (34–72) yrs 47.8% males	Cryoprecipitate (2 × 5 units, ~4.3 Fg). Intra- or post-op FIBTEM A20 of ≤12 mm triggered administration of more 10/5 units
Sabate et al., 2016 [37]	Spain	Aug 2012–Feb 2014	n = 48 54.5 (49–60) yrs 81.3% males	Prophylactic or therapeutic	Pre, intra, or post-op	Elective liver transplantation surgery	Pre-op plasma Fg level ≤ 2.9 g·L^−1^, or intra-op Fg levels < 1 g·L^−1^	1 g Fg until reaching plasma value of 2.9 g·L^−1^. (CSL Behring)	n = 44 57 (50–64) yrs 75% males	Placebo (0.9% saline solution)
Tanaka et al., 2013 [41]	USA	Jan 2011–May 2013	n = 10 71.3 (5.3) yrs 80% males	Therapeutic	Intra-op	Elective valve cardiac surgery with CPB	Visual bleeding scale of 2 or 3 §	4 g of Fg (RiaSTAP^®^, CSL Behring)	n = 10 66.1 (8.9) yrs 80% males	1 unit of apheresis PLTs

Data presented as mean (SD), mean (range), or median [IQR]. FC, fibrinogen concentrate; PLT, platelet; Fg, fibrinogen; MCF, maximum clot firmness of the fibrin-based clot; FIBTEM, fibrin-based thromboelastometry test; CPB, cardiopulmonary bypass. * Defined as combined coronary artery bypass graft (CABG) surgery and valve repair or replacement surgery, the replacement of multiple valves, aortic root reconstruction, or reconstruction of ascending aorta or aortic arch. † Fibrinogen concentrate dose (g) = (2.5 − [Plasma Fibrinogen Level at End of CPB, g/L]) × 0.07 × (1 − Hematocrit on CPB) × BodyWeight (kg). The target was 2.5 g·L^−1^. ‡ Fibrinogen concentrate dose (g) = (target FIBTEM MCF − actual FIBTEM MCF [mm]) − (body weight [kg]/70 × 0.5 g/mm). The target FIBTEM MCF was 22 mm. § Visual bleeding scale: 0 = excellent haemostasis (dry field), 1 = mild bleeding (oozing), 2 = moderate bleeding (controllable with applied pressure), and 3 = severe bleeding (multiple diffuse bleeding sites).

**Table 2:** Methodological quality assessment according to Cochrane risk of bias tool.

Items	Random Sequence Generation	Allocation Concealment	Performance	Detection	Attrition	Selective Reporting	Overall
Bilecen et al., 2017 [42]	●	●	●	●	●	●	●
Callum et al., 2019 [33]	●	●	●	●	●	●	●
Karlsson et al., 2009 [39]	●	●	●	●	●	●	●
Najafi et al., 2014 [38]	●	●	●	●	●	●	●
Rahe-Meyer et al., 2013 [34]	●	●	●	●	●	●	●
Rahe-Meyer et al., 2016 [35]	●	●	●	●	●	●	●
Ranucci et al., 2015 [40]	●	●	●	●	●	●	●
Roy et al., 2019 [36]	●	●	●	●	●	●	●
Sabate et al., 2016 [37]	●	●	●	●	●	●	●
Tanaka et al., 2013 [41]	●	●	●	●	●	●	●

● Green: low risk of bias; ● Red: high risk of bias; ● Yellow: unclear risk of bias.

## Supplementary Materials

The following supporting information can be downloaded at: https://www.mdpi.com/article/10.3390/jcm13123482/s1, Table S1: Preferred Reporting Items for Systematic Reviews and Meta-Analyses checklist; Table S2: Retrieval search strategy; Table S3: Summary of effect measures for fibrinogen concentrate administration outcomes in cardiovascular surgery studies; Figure S1: Forest plots for stroke or TIA according to comparator group. TIA, transient ischaemic attack; Figure S2: Forest plots for myocardial infarction according to comparator group; Figure S3: Forest plots for PE or DVT according to comparator group. PE, pulmonary embolism; DVT, deep venous thrombosis; Figure S4: Forest plots for stroke or TIA according to indication setting. TIA, transient ischaemic attack; Figure S5: Forest plots for myocardial infarction according to indication setting; Figure S6: Forest plots for PE or DVT according to indication setting. PE, pulmonary embolism; DVT, deep venous thrombosis; Figure S7: Forest plots for mortality according to indication setting; Figure S8: Forest plots for hospitalisation time according to comparator group; Figure S9: Forest plots for ICU time according to comparator group; Figure S10: Forest plots for serious adverse events according to comparator group; Figure S11: Forest plots for adverse events according to indication setting; Figure S12: Forest plots for serious adverse events according to indication setting.
