# Supplementary material for: Safety of Fibrinogen Concentrate in Non-Trauma and Non-Obstetric Adult Patients during Perioperative Care: Systematic Review and Meta-Analysis
Source: J Clin Med. 2024 Jun 14;13(12):3482. doi: 10.3390/jcm13123482 (PMC11204778; doi:10.3390/jcm13123482)
Supplement: Supplementary file 1 [file jcm-13-03482-s001.zip › Table S2 Search Strategy.pdf]

Table S2. Retrieval search strategy

**Query**	**Search: 03.03.2023**
**PubMed/Medline**	
#1	All fields: (((fibrinogen OR "factor I" OR "blood coagulation factor I" OR "coagulation factor I" OR "fibrinogen concentrate") **AND** (hemorrhag* OR bleed* OR "blood loss" OR "acquired hypofibrinogen*" OR hypofibrinogen* OR surger* OR perioperative OR "perioperative care")) **AND** (safety OR "patient* harm")) **AND** (adult*)
Filters	**Document type:** Classical Article, Clinical Study, Clinical Trial, Clinical Trial Protocol, Clinical Trial, Phase IV, Comparative Study, Controlled Clinical Trial, Corrected and Republished Article, Evaluation Study, Guideline, Introductory Journal Article, Meta-Analysis, Multicenter Study, Observational Study, Practice Guideline, Pragmatic Clinical Trial, Preprint, Published Erratum, Randomized Controlled Trial, Retracted Publication, Retraction of Publication, Review, Systematic Review, Validation Study<br>**Species:** Humans<br>**Age:** Adult: 19+ years.
**EMBASE**	
#1	('factor i'/exp OR 'factor i' OR 'blood coagulation factor i' OR 'coagulation factor i' OR 'fibrinogen concentrate'/exp OR 'fibrinogen concentrate') AND (hemorrhag* OR bleed* OR 'blood loss'/exp OR 'blood loss' OR 'acquired hypofibrinogen*' OR hypofibrinogen* OR surger* OR perioperative OR 'perioperative care'/exp OR 'perioperative care') AND ('safety'/exp OR safety OR 'patient* harm') AND adult*
**Scopus**	
#1	TITLE-ABS-KEY((((("factor i" OR "blood coagulation factor i" OR "coagulation factor i" OR "fibrinogen concentrate") AND (hemorrhag* OR bleed* OR "blood loss" OR "acquired hypofibrinogen*" OR hypofibrinogen* OR surger* OR perioperative OR "perioperative care")) AND (safety OR "patient* harm")) AND (adult*)))
Filters	**Document Types:** Article, Review, Conference Paper, Letter, Editorial, Note, Retracted
**Web of Science**	
#1	TS=(((fibrinogen OR "factor I" OR "blood coagulation factor I" OR "coagulation factor I" OR "fibrinogen concentrate") AND (hemorrhag* OR bleed* OR "blood loss" OR "acquired hypofibrinogen*" OR hypofibrinogen* OR surger* OR perioperative OR "perioperative care")) AND (safety OR "patient* harm")) AND TS=(adult*)
**Cochrane Database of Systematic Reviews**	
#1	(((fibrinogen OR "factor I" OR "blood coagulation factor I" OR "coagulation factor I" OR "fibrinogen concentrate") **AND** (hemorrhag* OR bleed* OR "blood loss" OR "acquired hypofibrinogen*" OR hypofibrinogen* OR surger* OR perioperative OR "perioperative care")) **AND** (safety OR "patient* harm")) **AND** (adult*)
**Cochrane Central Register of Controlled Trials**	
#1	(((fibrinogen OR "factor I" OR "blood coagulation factor I" OR "coagulation factor I" OR "fibrinogen concentrate") **AND** (hemorrhag* OR bleed* OR "blood loss" OR "acquired hypofibrinogen*" OR hypofibrinogen* OR surger* OR perioperative OR "perioperative care")) **AND** (safety OR "patient* harm")) **AND** (adult*)
