# Supplementary material for: Safety of Fibrinogen Concentrate in Non-Trauma and Non-Obstetric Adult Patients during Perioperative Care: Systematic Review and Meta-Analysis
Source: J Clin Med. 2024 Jun 14;13(12):3482. doi: 10.3390/jcm13123482 (PMC11204778; doi:10.3390/jcm13123482)
Supplement: Supplementary file 1 [file jcm-13-03482-s001.zip › Table S3.pdf]

**Table S3.** Summary of effect measures for Fibrinogen concentrate administration outcomes in cardiovascular surgery studies

| Outcome | Odds Ratio | 95% CI | $I^2$ (%) | *p*-value for subgroup differences | |
|---|---|---|---|---|---|
| | | | | Fibrinogen vs comparator | Prophylactic vs therapeutic |
| **Thromboembolic events** | 0.72 | 0.47 – 1.11 | 0 | 0.95 | 0.48 |
| **Stroke/TIA** | 0.90 | 0.51 – 1.61 | 0 | 0.93 | 0.96 |
| **Myocardial Infarction** | 0.98 | 0.35 – 2.69 | 0 | 0.50 | 0.57 |
| **PE/DVT** | 0.66 | 0.26 – 1.63 | 0 | 0.82 | 0.83 |
| **Overall mortality** | 0.93 | 0.5 – 1.74 | 11 | 0.44 | 0.55 |
| **Adverse events** | 0.80 | 0.61 – 1.04 | 0 | 0.63 | 0.91 |
| **Serious adverse events** | 0.90 | 0.68 – 1.18 | 0 | 0.56 | - |
| | **Mean Difference** | | | | |
| **Hospitalization time** | -0.64 | -1.40 – 0.11 | 0 | 0.38 | - |
| **ICU time** | -0.04 | -0.51 – 0.42 | 3 | 0.36 | - |

CI, confidence interval; DVT, deep venous thrombosis; ICU, intensive care unit; PE, pulmonary embolism; TIA, transient ischemic attack.
