# Supplementary figures and images for: Safety of Fibrinogen Concentrate in Non-Trauma and Non-Obstetric Adult Patients during Perioperative Care: Systematic Review and Meta-Analysis
Source: J Clin Med. 2024 Jun 14;13(12):3482. doi: 10.3390/jcm13123482 (PMC11204778; doi:10.3390/jcm13123482)

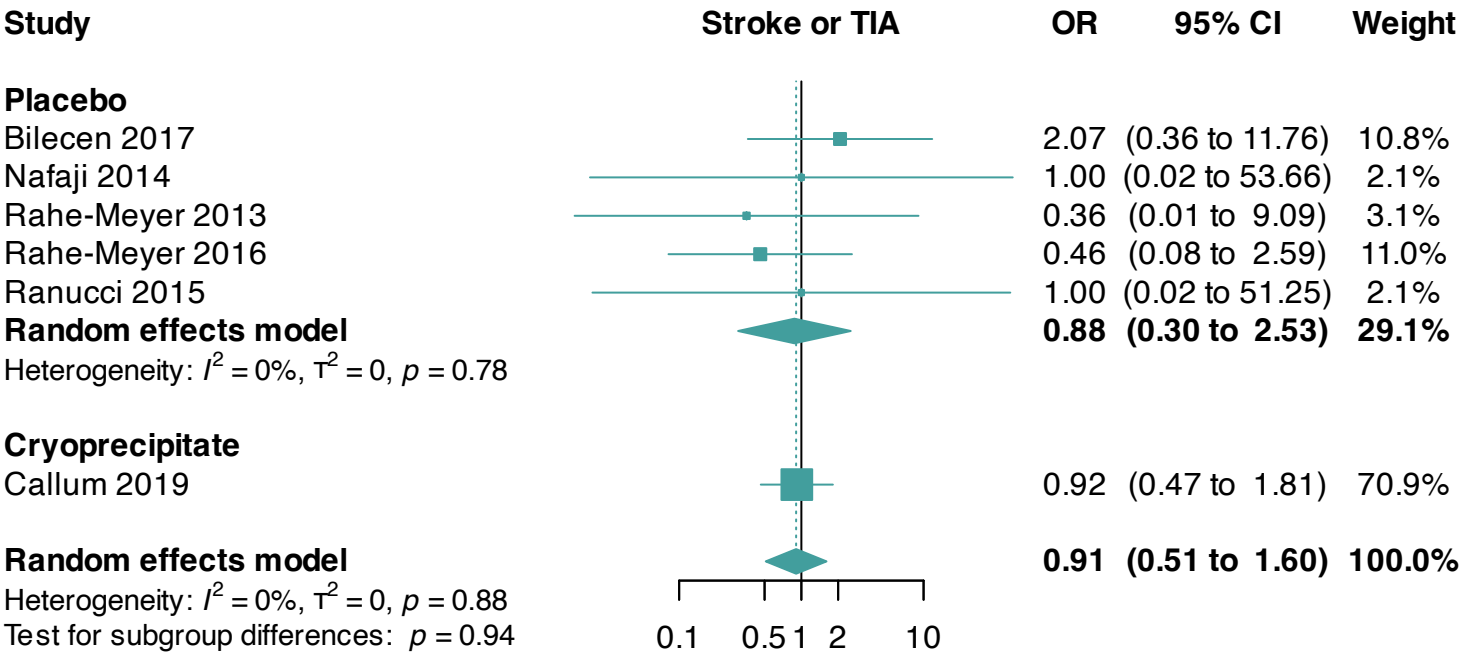

Supplement: Supplementary file 1 [file jcm-13-03482-s001.zip › Figure S1.pdf]

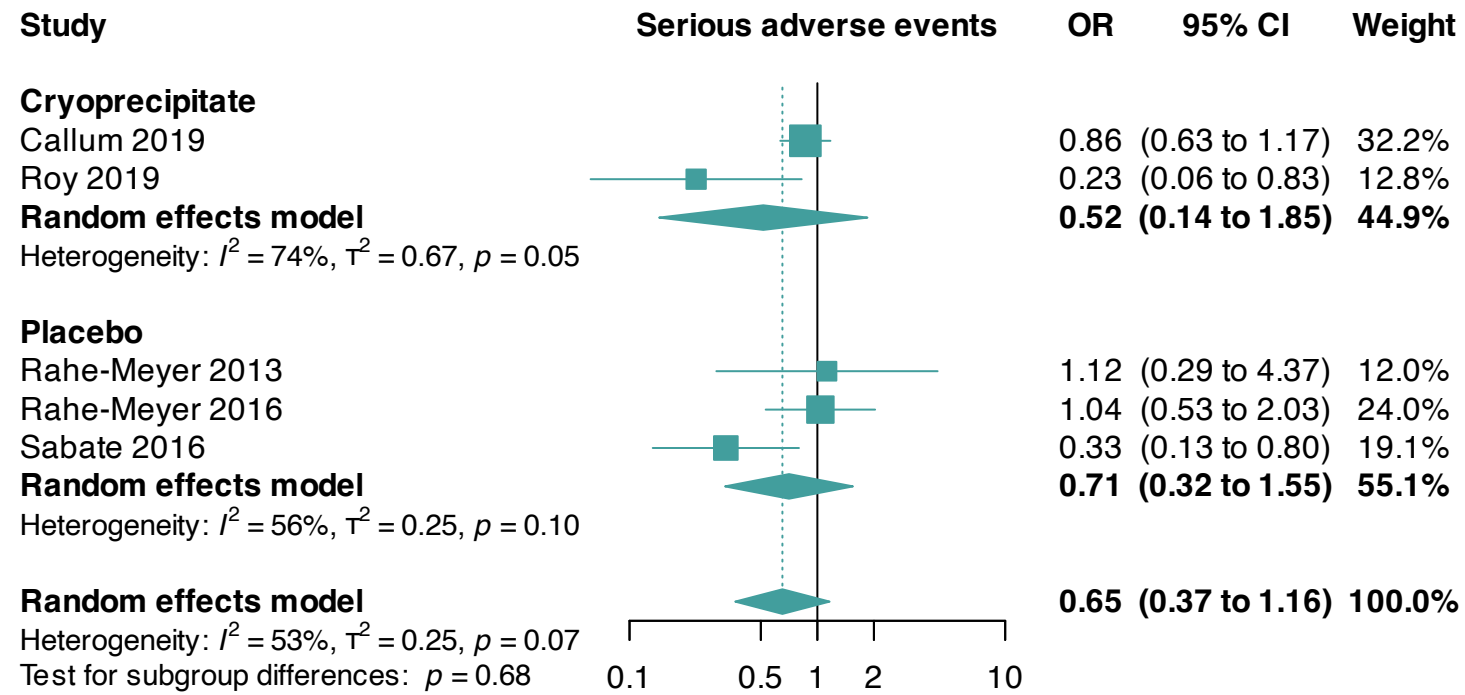

Supplement: Supplementary file 1 [file jcm-13-03482-s001.zip › Figure S10_Serious adverse events 1.pdf]

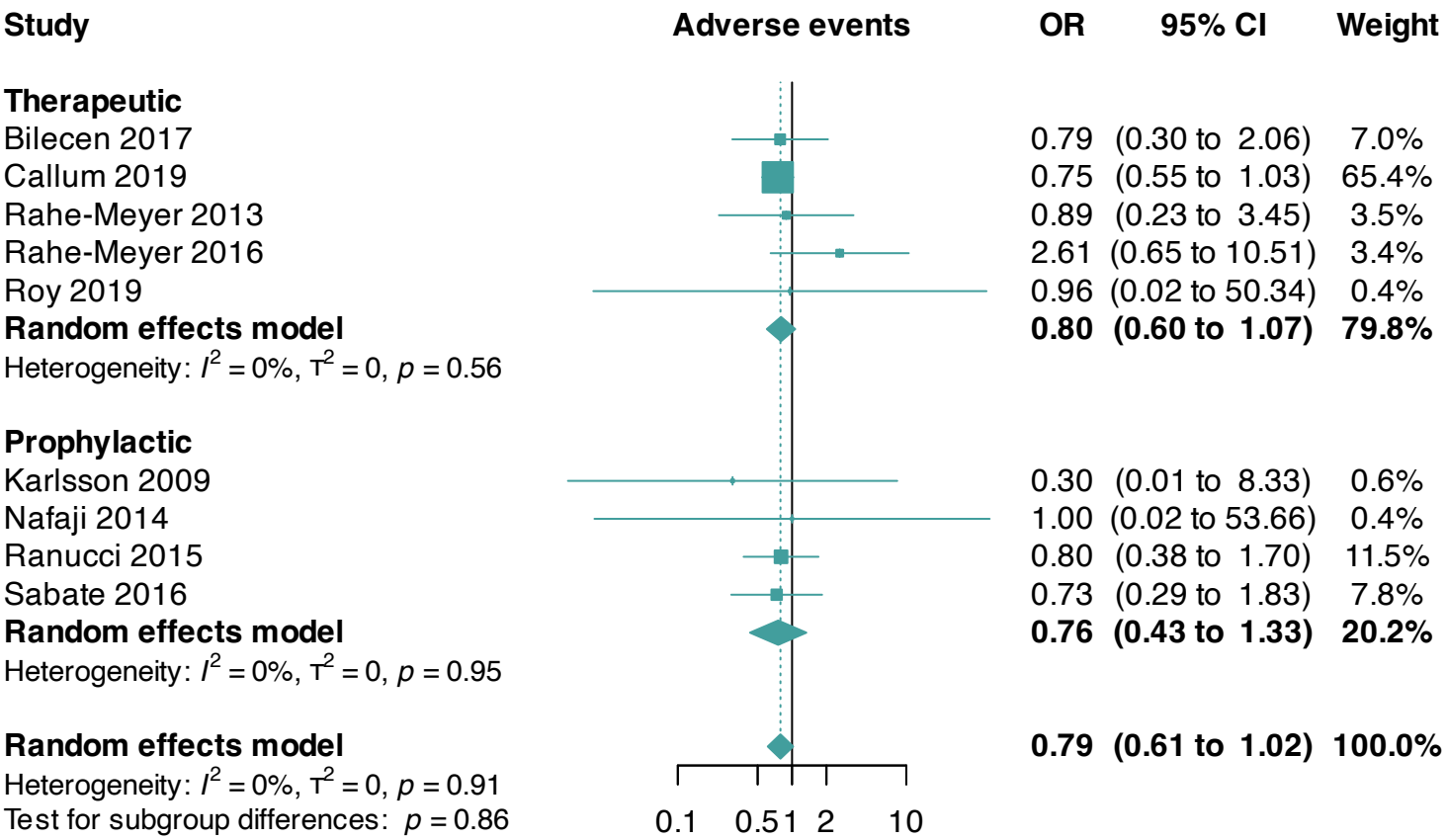

Supplement: Supplementary file 1 [file jcm-13-03482-s001.zip › Figure S11_Adverse events 2.pdf]

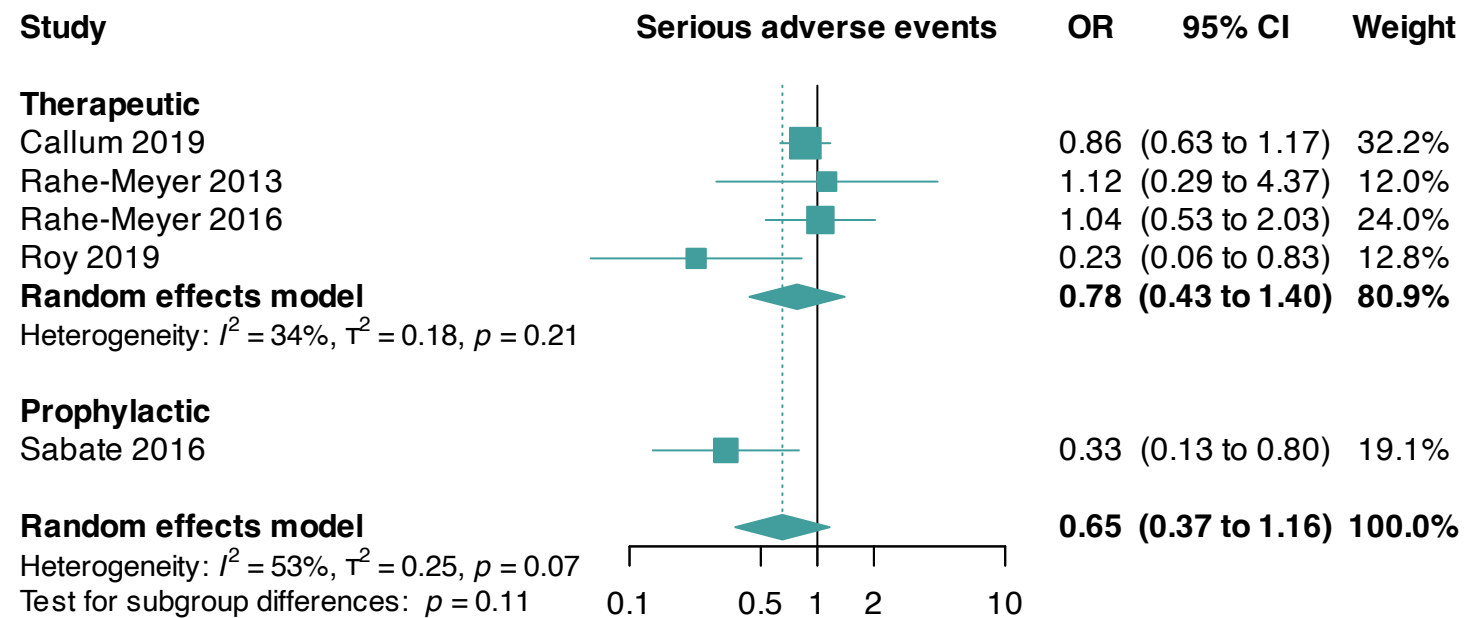

Supplement: Supplementary file 1 [file jcm-13-03482-s001.zip › Figure S12_Serious adverse events 2.pdf]

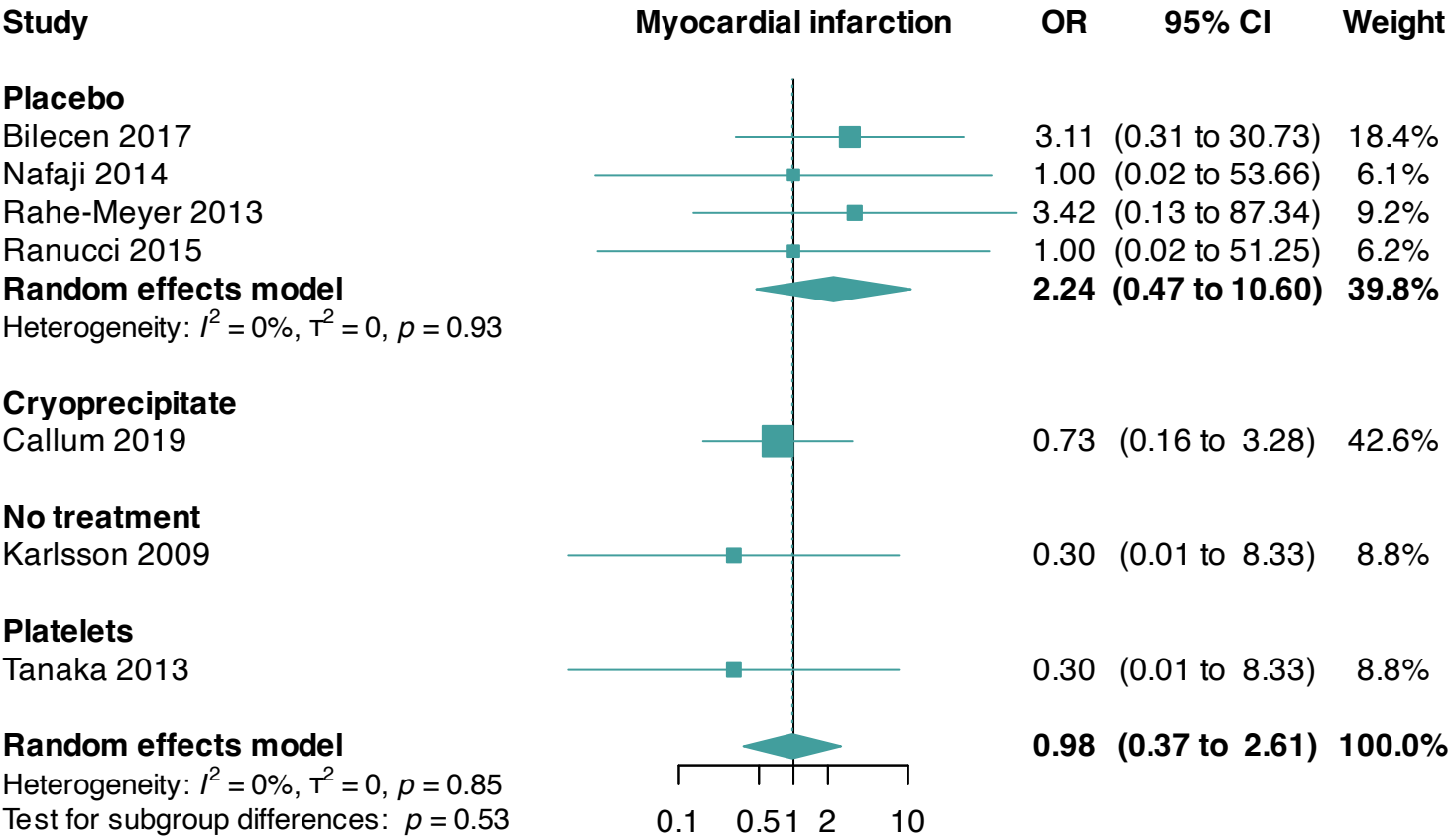

Supplement: Supplementary file 1 [file jcm-13-03482-s001.zip › Figure S2.pdf]

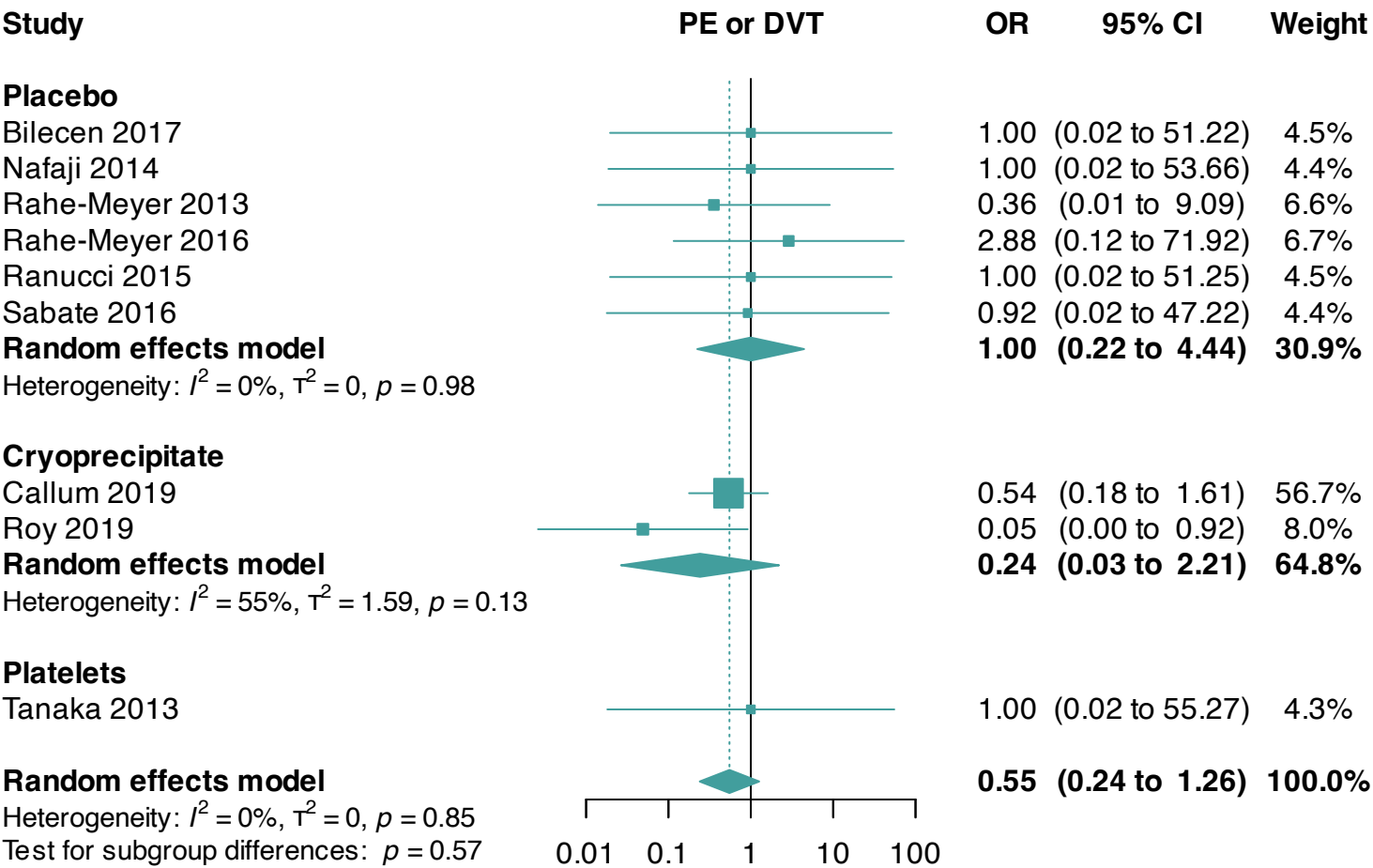

Supplement: Supplementary file 1 [file jcm-13-03482-s001.zip › Figure S3.pdf]

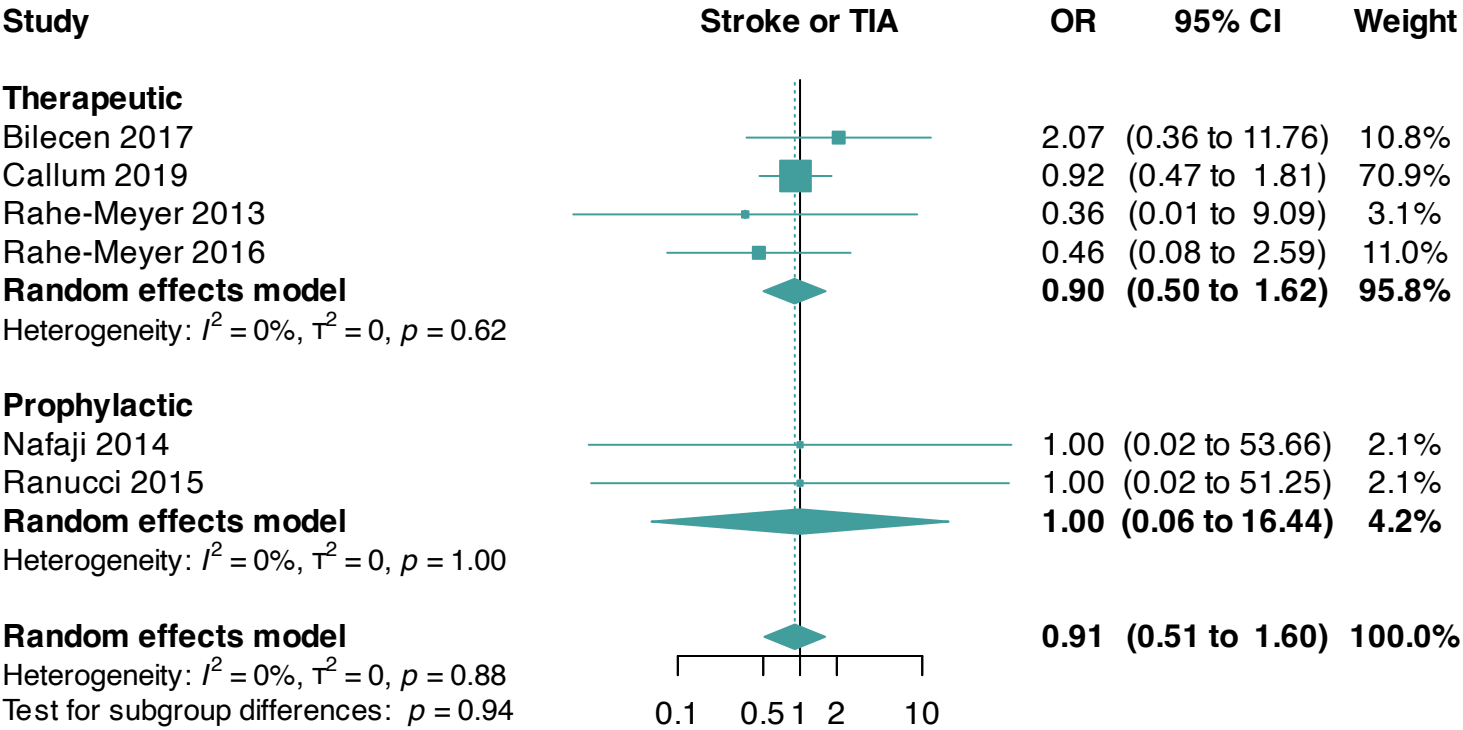

Supplement: Supplementary file 1 [file jcm-13-03482-s001.zip › Figure S4_Stroke 2_.pdf]

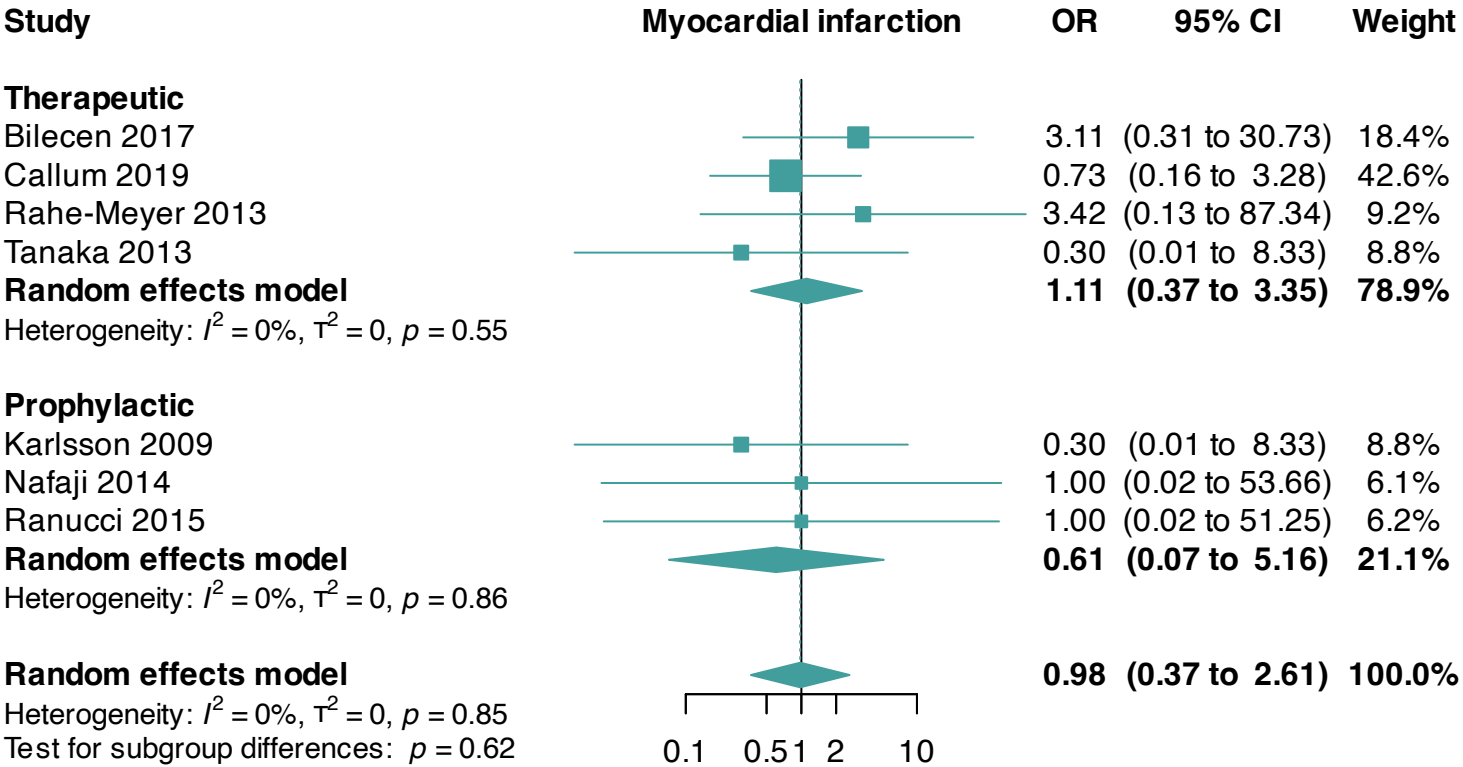

Supplement: Supplementary file 1 [file jcm-13-03482-s001.zip › Figure S5_Myocardial infarction 2_.pdf]

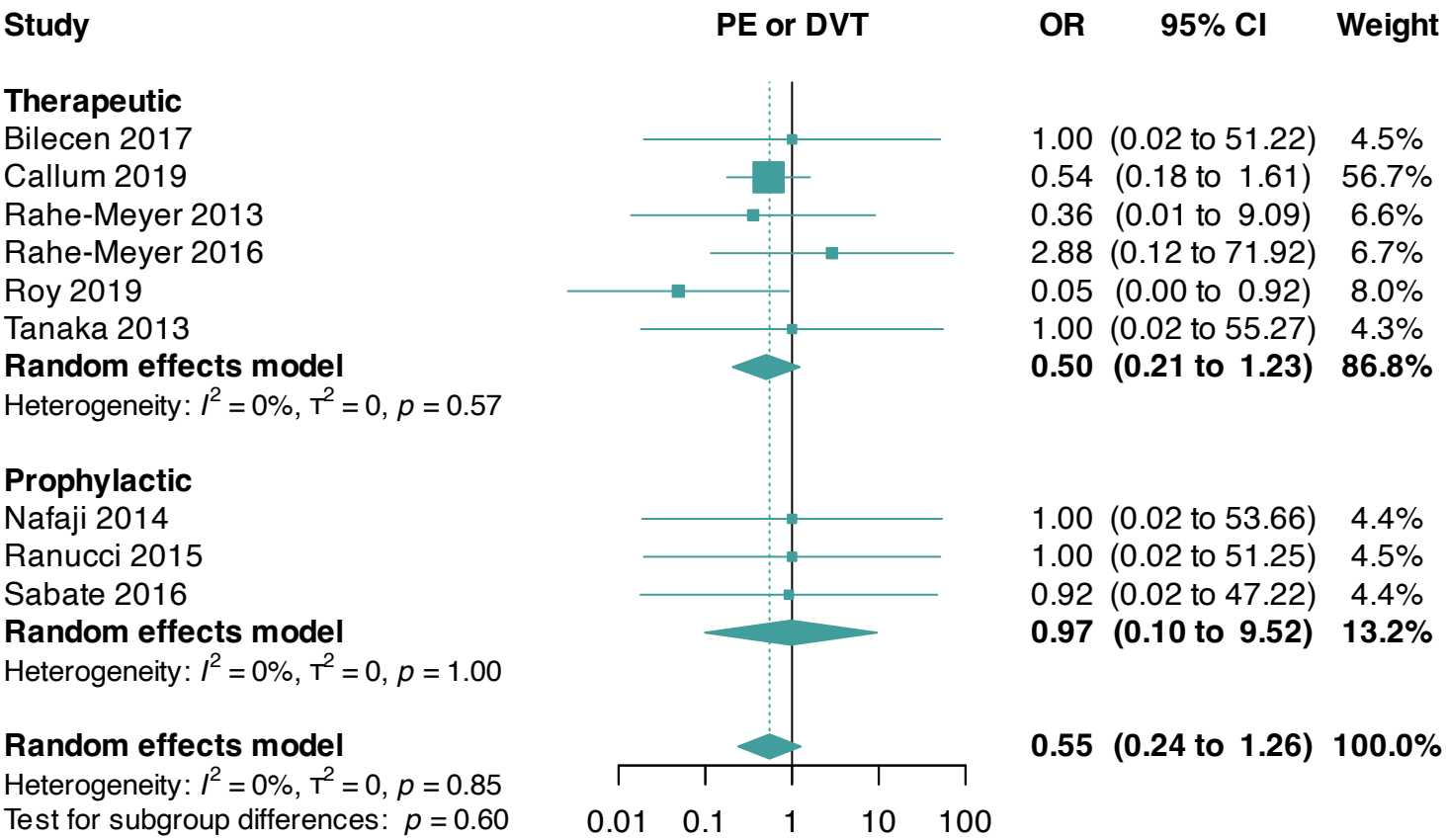

Supplement: Supplementary file 1 [file jcm-13-03482-s001.zip › Figure S6_PE 2.pdf]

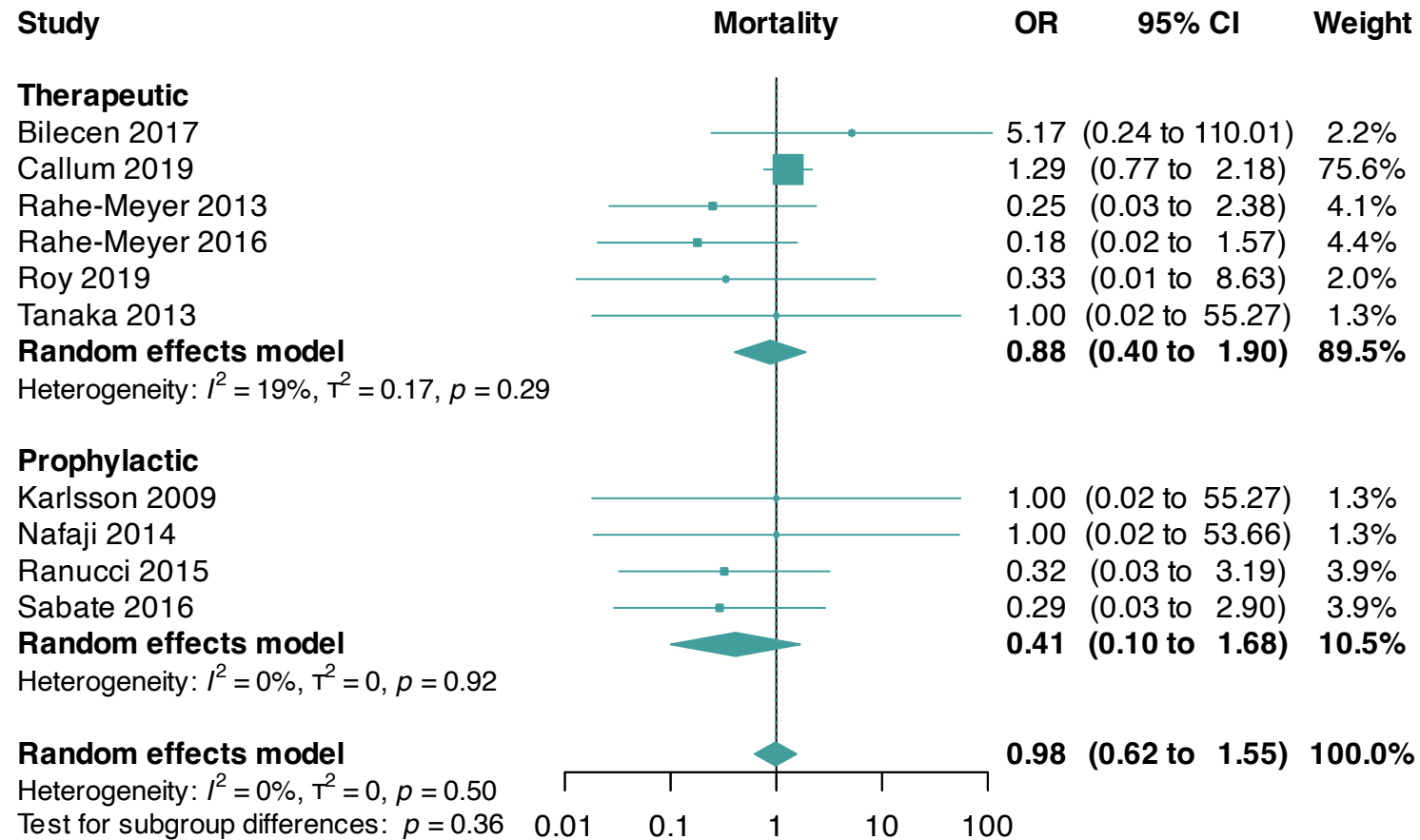

Supplement: Supplementary file 1 [file jcm-13-03482-s001.zip › Figure S7_Mortality 2.pdf]

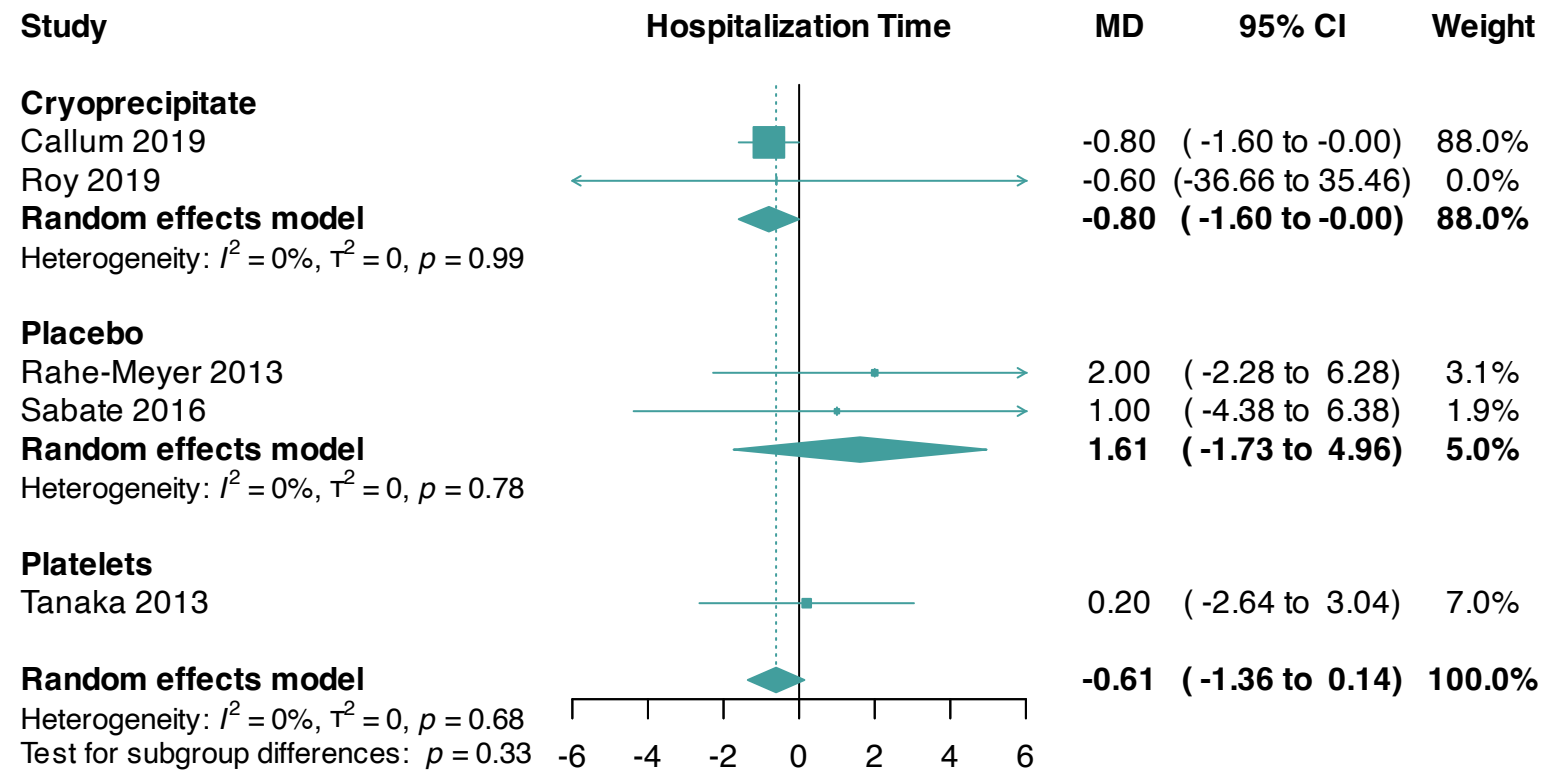

Supplement: Supplementary file 1 [file jcm-13-03482-s001.zip › Figure S8_Hospitalization Time 1.pdf]

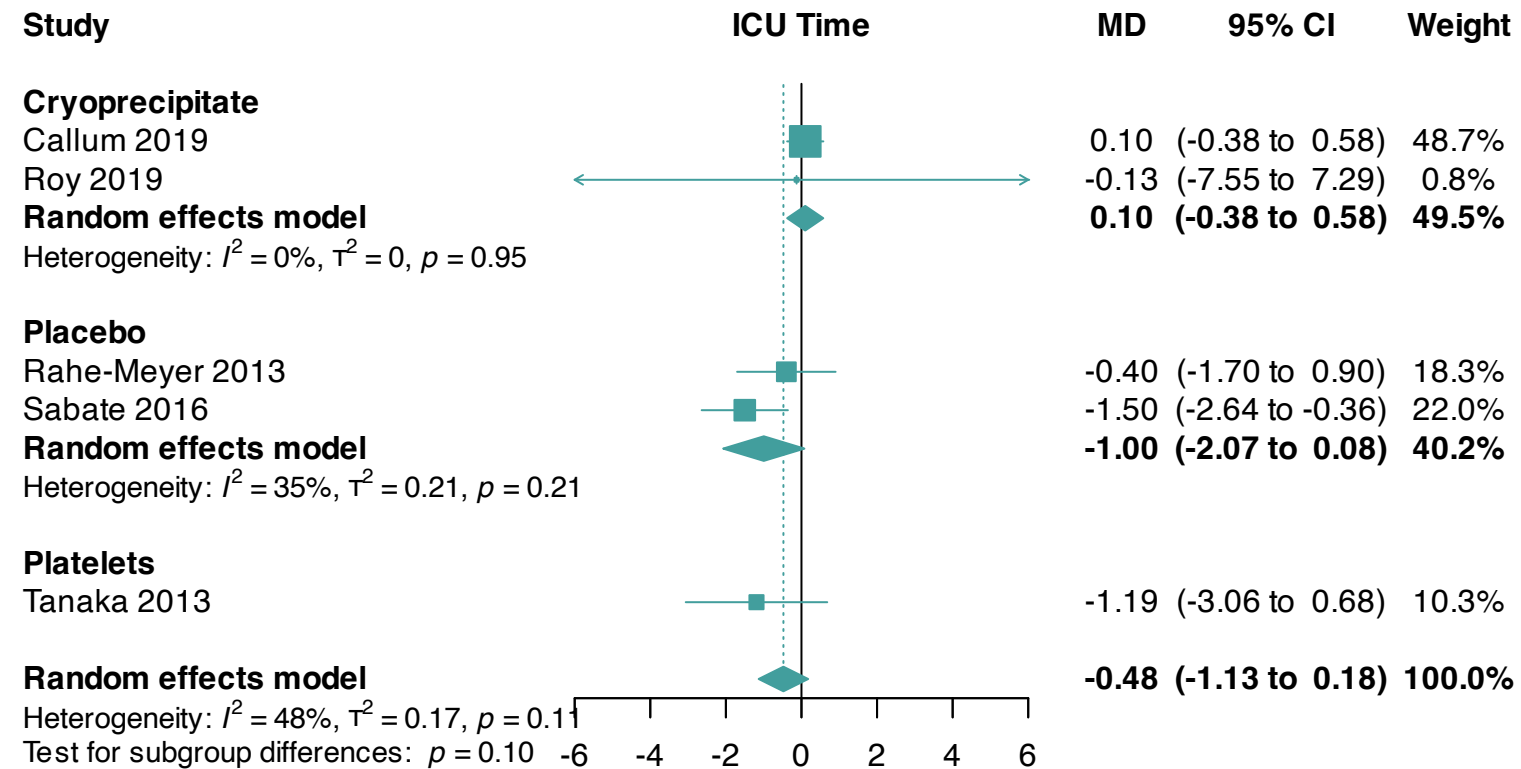

Supplement: Supplementary file 1 [file jcm-13-03482-s001.zip › Figure S9_ICU time 1.pdf]
